# Biocompatibility of nano/micro-sized pyrophyllite particles by pulmo, liver, kidney and gastric mucosis cells

**DOI:** 10.1007/s10856-024-06793-z

**Published:** 2024-06-17

**Authors:** Smiljana Paraš, Jovana Paspalj, Karima Baghdad, Ognjenka Janković, Ranko Škrbić, Radoslav Gajanin, Pascale Massiani, Franck Launay, Suzana Gotovac Atlagić

**Affiliations:** 1 University of Banja Luka, Faculty of Natural Sciences and Mathematics, Mladena Stojanovića 2, 78000 Banja Luka, Bosnia and Herzegovina; 2 Sorbonne Université, Laboratoire de Réactivité de Surface (LRS – UMR 7197 CNRS), 4 place Jussieu, 72252, Paris Cédex 05, France; 3 University of Oran 1-Ahmed Ben Bella, Faculty of Exact and Applied Sciences, Materials chemistry laboratory-LCM, Department of Chemistry, P.O. Box 1524, Oran, 31005 Algeria; 4 University of Banja Luka, Faculty of Medicine, Save Mrkalja 14, 78000 Banja Luka, Bosnia and Herzegovina

## Abstract

Pyrophyllite is the least studied natural clay in terms of its potential in biomedical applications, although there are many deposits of this aluminosilicate around the world. Genotoxicity study was performed in vitro for this mineral. Subsequently, Wister rats were exposed to the pyrophyllite micronized to below 100 µm. After the exposure period, histology of the lung, liver, kidney and gastric tissues were performed, followed by the stereological and hematological analysis. The physicochemical analyses revealed typical XRD characteristics of pyrophyllite clay with particle-size distribution ranging 50 nm–100 μm with stable mineral composition and unique buffering property to pH around 8. The results showed that there were no cytotoxic effects on to THP-1 cells, or genotoxicity of pyrophyllite measured by the Comet assay. In vivo studies are accompanied by the thorough physicochemical characterization of the micronized pyrophyllite. Histology of the lung tissue proved presence of an inflammatory reaction. On the other hand, gastric tissue has shown the selective accumulation of nanoparticles in enterocytes of the stomach only, as supported by ultrastructural analysis. Liver and kidney tissues have shown tolerability for pyrophyllite particles. The results give directions for further comprehensive studies of potential biomedical applications of the pyrophyllite.

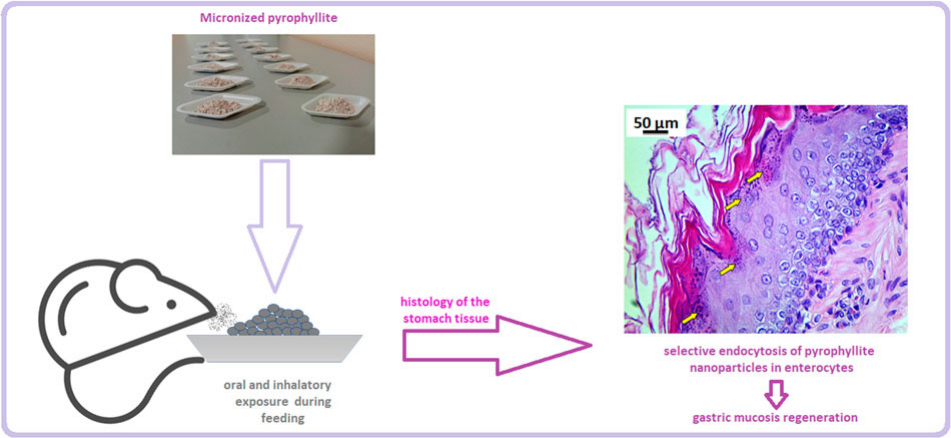

## Introduction

Natural aluminosilicates are often considered as the useful supplements to the human and animal food. When it comes to animal feed, most of the literature discusses their potential for adsorption of toxic organic molecules such as mycotoxins [1]. Other studies strongly evidenced that the use of 2% of bentonite supplement in the food for *Escherichia coli*-infected piglets’ has beneficial effects on regeneration process of small intestines mucosa [2]. Gonzales et al. have shown that also the diosmectite clay has the regenerative effects on colon in vivo in rats. The mechanism was proved the improvement of mucin secretion in the presence of clay. Such evidences are very promising in the search for treatment of the most severe intestinal illnesses such as Crohn’s disease or ulcerative colitis [3]. In humans, some mineral clays were even studied clinically as the osteoarthritis treatments in the form of orally consumed capsules [4, 5]. Pyrophyllite is a natural clay with very high similarity to the talc. Talc is widely used in pharmaceutical and galenic products, which makes the pyrophyllite also a promising candidate in the similar areas of applications. For example, pyrophyllite could be studied as a carrier for different kinds of medicaments. This natural clay is rich in Fe, Ca, Mg, K, Zn and other essential elements. Pyrophyllite has gentle alkaline pH and shows alkaline buffering properties, so the medical application potential of this mineral has to be additionally studied. Pyrophyllite exists on multiple locations around the world with slight variations in its composition [6].

Health applications of pyrophyllite mainly rely on an acid buffering effect and an anion exchange property. Studies to date have shown that pyrophyllite particles do not enter enterocytes but remain on the surface of the mucosa and bind gastric acid ions. Natural pyrophyllite was suggested to have barrier properties similar to those of gastric mucous, and to afford mucosal protection by its ability to maintain or mimic the barrier properties of gastric mucous gel [7].

The purpose of present study is to explore the potential for biomedical applications of the pyrophyllite since there are not enough data in this study field. Study of the toxic metal accessibility from pyrophyllite was performed, to access its health safety, followed by detailed physicochemical characterizations. Next, in vivo study in Wister rats, the animals were exposed to the pyrophyllite orally and inhalatory. There is no comprehensive study including health safety, genotoxicity, and biological tolerability of this mineral. Therefore, depending on the characteristics of the pyrophyllite, results should give directions for the future research on application of pyrophyllite in animal food, galenic products and biomedical industry in general.

## Results and discussions

### Physicochemical characterization of the pyrophyllite

The XRF analysis showed that the highest percentage of the composition is based on silicon (32.21%) and aluminum (9.76%) which is expected for this type of aluminosilicate. Regarding the other elements, notably high was the percent of calcium (5.87%) which must be considered in discussions on the potential medical applications studied here (Table 1). Next by abundance were the iron (1.04%), potassium (0.74%) and magnesium (0.39%) while, titanium and zinc were found in traces.

**Table 1:** Results of the x-ray fluorescence (XRF) spectroscopy measurements (presence of metals, limit of detection above 0.3 weight %)

Sample	XRF (chemical composition in weight %)							
	Mg	Al	Si	K	Ca	Ti	Fe	Zn
Raw pyrophyllite	0.39	9.76	32.21	0.74	5.87	0.11	1.04	0.13

**Fig. 1:**
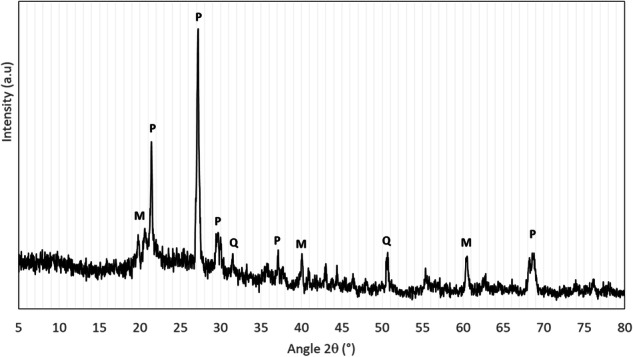
X-ray diffractogram of the pyrophyllite (M-mica, Q-quartz, P-pyrophyllite)

Diffractogram of the pyrophyllite sample shows all typical pyrophyllite peaks at around 21, 27, 29, 37, 68 degrees (Fig. 1). Also, the quartz peaks are visible at values of 31 and 51, which could be expected since often quartz follows the soft aluminosilicates in their structure. This is the case for pyrophyllite as well, and quartz is usually found in its structure [8, 9]. Some of the peaks can be determined as mica (K_2_O) which agrees with the metal contents determined by XRF. XRD pattern of pyrophyllite proved the presence of amorphous, which influences its crystallinity. The typical peaks of pyrophyllite and kaolinite can be noticed. Some organic impurities such as nitrogen, carbon gasses as well as crystalline water could be expected to be trapped between the planes.

Metal migration from the pyrophyllite is rather low, particularly in the pH areas relevant for the human environment (soil, skin). Most similar to the present study is the report of Chen et al., who used Sierra Mountains mineral clays and studied the bioaccessibility of the toxic metals from this clay in order to assess the potential risks [10]. Like in their work, final judgment about the health safety of the pyrophyllite studied here must be considered depending on the appropriate legislation of the country and area of application where it would be used. Some of the potentially critical values are given in bold, such as leaching of the Cu, Cr, Pb, As and Al under strongly acidic conditions (Supplementary Table S1).

One unexpected pH property was observed during this experiment. Namely, pyrophyllite shows the capability of neutralizing the acidic solutions. The most pronounced change can be observed in the gray-colored area of the pH (Supplementary Table S2). In short, 1 g of pyrophyllite is capable to completely neutralize 100 mL of a strongly acidic solution (e.g. from pH 2 to pH 6.86). This is not only neutralization but is also adjustment into the mildly alkaline region of pH. This tendency deserves attention since it could have great potential in wastewater and any other acidic waste treatment.

### Genotoxicity

**Fig. 2:**
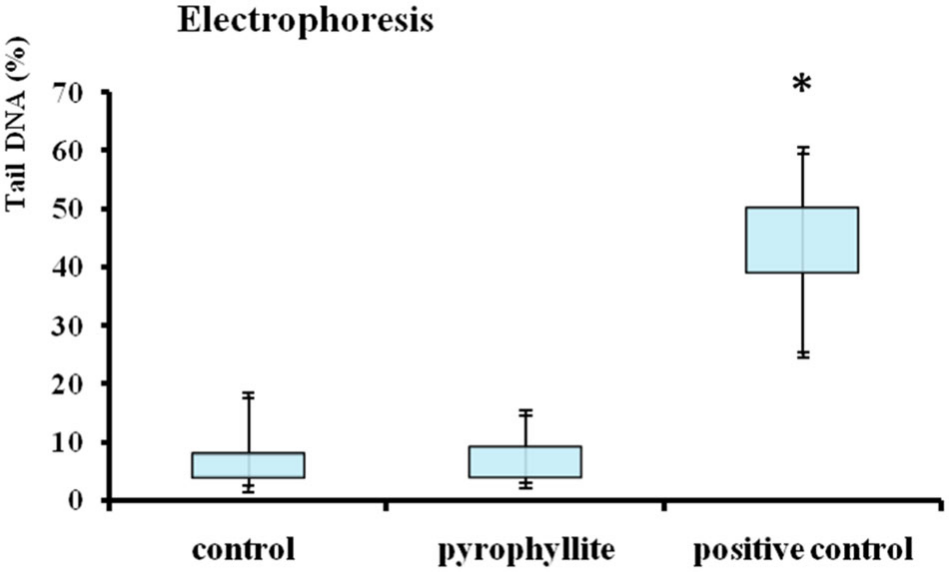
Electrophoresis after Comet assay, control, pyrophyllite and positive control (*n* = 12). Asterisk denote the significant differences with respect to the untreated control cells (*p < 0.001; one-ay ANOVA, Dunnett’s test)

**Fig. 3:**
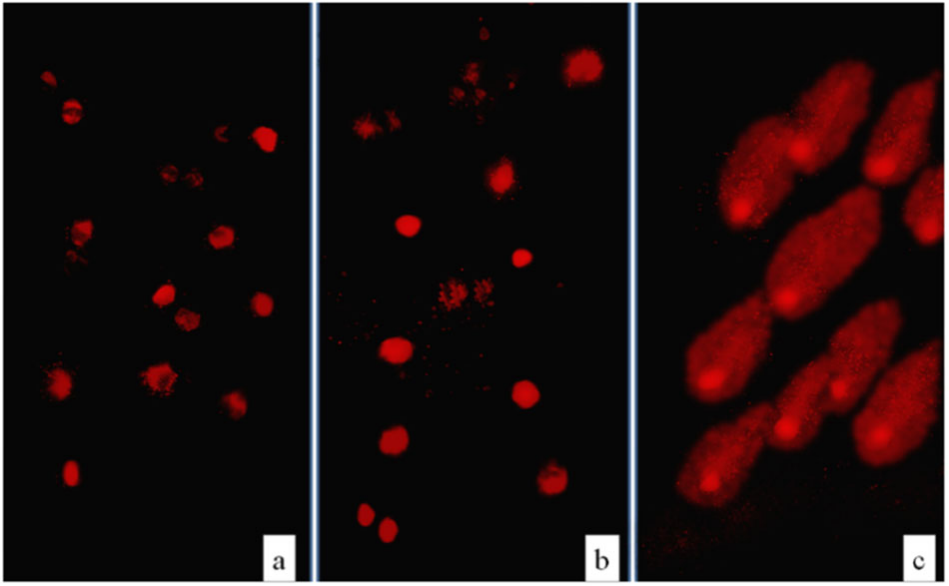
Comet assay. Images of comets tail for **a** control group, **b** experimental group and **c** positive control

The results of Trypan blue exclusion assay indicated that pyrophyllite was not cytotoxic to THP-1 cells after 1 h exposure. In all samples treated with pyrophyllite cell viability was over 90%. The results of the Comet assay showed that pyrophyllite was not genotoxic to THP-1 cells (Fig. 2). When compared to the control (Fig. 3a), there is no comet tail (Fig. 3b) in cells which are treated with pyrophyllite, which means that there is no damaged DNA (broken fragments) that would migrate from the “head” during the electrophoresis (Fig. 2), like in the case of positive control, as it can be seen in Fig. 3c.

Extensive literature search did not show any article dealing with the genotoxicity of the pyrophyllite as such. Therefore, the present results could open this subject to wider research. As it is the most widely used methodology, for the genotoxicity test, the elements present in largest percentages were used in the Comet assay. These were silicon, aluminum and calcium as can be seen from the XRF data in Table 1. This study aimed to evaluate the genotoxicity of pyrophyllite in vitro, which is very important for its potential application in medicine. Genotoxicity study was based on THP-1 cells using a Comet assay. This test showed that the pyrophyllite extract did not cause changes within the cells at the DNA level and did not damage DNAs in the THP-1 cells. The lack of DNA damage in THP-1 cells was significantly reliable compared to the positive control. This is consistent with previous studies that also reported a lack of genotoxicity of aluminum from aluminum chloride, calcium from biphasic calcium phosphate, and silicon from silicon nanoparticles [11–13]. Although these authors, as mentioned above, did not deal with the pyrophyllite, their results are the closest literature data which can be used for evaluation the genotoxicity of these major components of pyrophyllite.

The test of the aluminosilicate particles genotoxicity is harder to perform, due to the limited literature data. For example, Sharma et al. have studied the genotoxic of montmorillonite particle suspensions of two classes: crude suspension and the sieved to below <0.2 µm [14]. On the other hand, Li et al. have studied also natural montmorillonite, however modified in a way that it was used to exfoliate the nanosilicate platelets. The polyamine salt served as the exfoliator resulting in plates smaller than 80 nm in diameter. These particles also caused no mutation in DNA of Chinese Hamster Ovary cells [15].

### Blood testing

#### Hematological analyses

All values of the parameters of blood analysis were similar in the animals exposed to pyrophyllite and control group, except for the number of white blood cells and neutrophil granulocytes (Supplementary Table S3). The number of white blood cells of the group of animals exposed to pyrophyllite (10.17 ± 2.13) was significantly higher in relation to the number of white blood cells in the group of control animals (5.65 ± 1.25), *p* < 0.001. Also, the number of neutrophil granulocytes and their percentage in the total number of all three types of granulocytes were significantly increased (*p* < 0.001) in the group of animals exposed to pyrophyllite compared to controls. Most of the authors agree that the range of normal white blood cell counts in rats is very wide [16, 17]. In the blood of rats exposed to pyrophyllite, there was an increase in the number of white blood cells above the upper limit and this increase was significant compared to the number of white blood cells in control rats. The increase in the mean value of the number of white blood cells and neutrophil granulocytes in rats exposed to pyrophyllite indicates the existence of some inflammatory process. Neutrophil granulocytes and macrophages combine and together participate in the endocytosis and therefore neutralization of pyrophyllite particles by phagocytosis. This results is in excellent agreement with the study of Sato et al. [18–20]. They studied the effects of talc on hamster lung tissue. They also found the phagocytosis of talc by the macrophages, which is significant since pyrophyllite is a hydrated silicate of aluminum while talc is hydrated silicate of magnesium, differing only in one ion, but having the same crystal structure. This conclusion can also be partially compared to similar relation found in the studies where rats were exposed to amorphous silica and nickel oxide nanoparticles, leading to an increase in the number of leukocytes [19, 20]. Blood of the rats exposed to pyrophyllite had a lower number of erythrocytes than the blood of the control group; however, this reduction was not significant. Decrease in the erythrocytes number in the blood of rats exposed to pyrophyllite, was most probably caused by their increased infiltration in the tissues of the lungs, kidneys, spleen, and liver. The same conclusion was reached by the study on influence of calcium-containing implants on rat hematology [21]. A new type of the dental filling was tested in the standard dental filling procedure and as the under-the-skin implant made of nanomaterial based on calcium aluminate and calcium silicate in order to study its toxicity. The same change in the number of erythrocytes like in the present paper was measured in the blood of rats with implants. Next comparison can be an in vivo study in rats, on the effect of Eudragit Retard L (ERL) nanoparticles from the oral suspension [22]. This study showed that the number of erythrocytes decreased in the blood of rats after two days of exposure, using this non-biodegradable copolymer nanoparticle, with a larger size (above 300 nm diameter). However, three weeks into exposure to the oral suspension of ERL nanoparticles, the number of erythrocytes returned to normal. Authors noted that the most important factor of nanoparticles effect on the blood in rats was the duration and frequency of exposure.

#### Biochemical analyses

Analysis of biochemical parameters showed that the values of total protein and potassium increased significantly in the group of rats exposed to pyrophyllite compared to the control group by *p* < 0.001. Furthermore, in animals exposed to pyrophyllite, the values of creatinine and potassium were higher than the reference range values. All other values of the parameters of the biochemical analysis of blood did not change in the group of rats exposed to pyrophyllite in relation to the control ones and were within the reference range of normal values (Supplementary Table S4).

The increase in total proteins and urea in the blood of rats exposed to pyrophyllite in relation to the control ones is in accordance with the results of Espinosa et al. [23]; in which the total protein in the blood of rats increases significantly in the treatment with silver nanoparticles in relation to control values. The explanation for the increase in total protein is the increased number of white blood cells in the blood and macrophages in the body fluids of the tissues of rats that were exposed to nanoparticles. The increase in creatinine in the blood of rats exposed to pyrophyllite is the same as [24]. The authors explain the increase in creatinine as the reason for the burdened work of the kidneys. In rats, the kidneys filter exposed nanoparticles from the blood. A significant increase in potassium ions in the blood of rats exposed to pyrophyllite is the same as in the work of the Wim ; rats were treated on the basis of copper with calcium nanoparticles [25]. The multiplied concentration of potassium is justified by the position of its ions on the aggregates of nanoparticles of pyrophyllite from which it is easily separated [26]. The leaching behavior of the potassium contained in different types of aluminosilicates rocks is a well-known phenomenon and it is a desirable characteristic [27]. Since the stomach contains at least 0.1 M of HCl, it is reasonable to conclude that potassium from pyrophyllite (Table 1) has migrated in the stomach environment and later had been easily absorbed to the blood from intestines.

### Histological and stereological analyses

#### Lung tissue

**Fig. 4:**
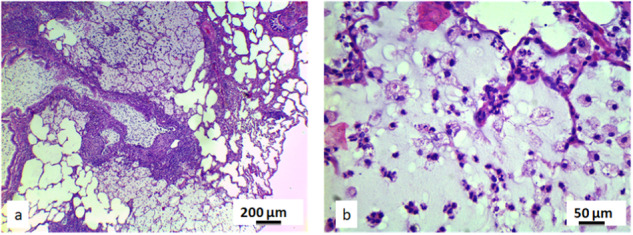
Micrographs of the histological cross-section of the lung in exposed to the pyrophyllite, H&E: inflammation process with pneumonia are visible at two different magnifications, healthy tissue occluded with inflammatory tissue, magnification 20× (**a**), inflammatory cells, magnification, 100× (**b**)

**Fig. 5:**
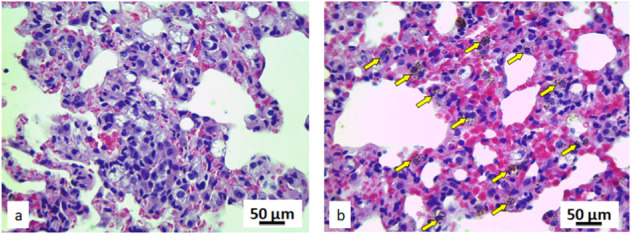
Micrographs of the histological cross-section of the lung, H&E, magnifications 100×: control (**a**) and exposed to pyrophyllite, yellow arrows show macrophages (**b**)

**Fig. 6:**
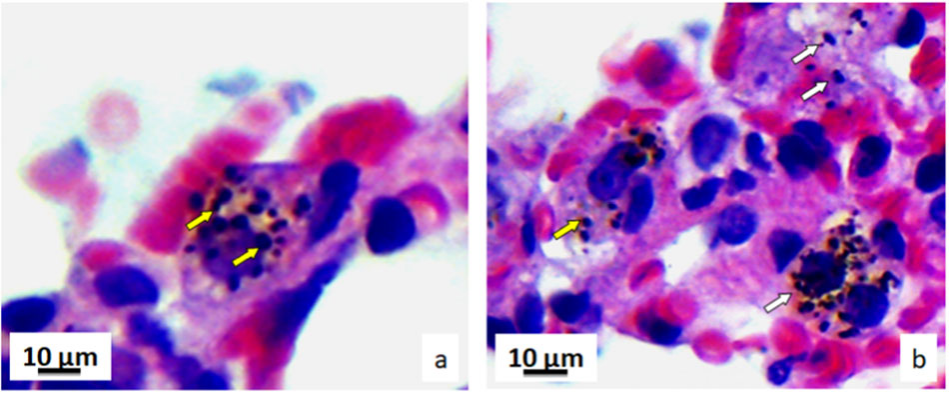
Micrographs of the histological cross-section of the lung exposed to pyrophyllite, Mallory-Azan coloured, magnification ×500: yellow arrows pointing at accumulations of the pyrophyllite particles in cytoplasm (**a**); white arrows pointing at accumulations of the pyrophyllite particles in pneumocytes and yellow arrows at macrophages (**b**)

Macrophages of all the animals exposed to the pyrophyllite (*n* = 10) have shown the presence of the vacuolized pyrophyllite particles. However, 5 out of these 10 animals have also had a clear presence of the local lung inflammation. Morphology of the remaining tissue in exposed animals, was not different from the control; all pneumocytes, alveolus and the blood vessels were of normal structure. For all these exposed animals, no presence of pathological changes such as cysts, fibrosis, and loss of pneumocytes, lymphocyte aggregates and necrosis were detected. Further histological and cytological analysis of the lung tissue of the rats, was based on the comparison of the 21 stereological parameters (Supplementary Table S5). Volume density of the epithelium lung cells and macrophages; number of the macrophages; numerical density of the macrophages and the erythrocytes (*p* < 0.05) have shown a significant increase in the lung tissue of the exposed rats. Five out of ten animals exposed to the pyrophyllite had bilateral but localized pneumonia (Fig. 4a, b). Lung tissue of all the rats exposed to pyrophyllite contained plenty of macrophages (Fig. 5b), while, in the case of rats from the control group, there were no macrophages whatsoever (Fig. 5a). This clearly explains the high white blood cell count detected in hematological analysis which pointed out at inflammation, and there is no doubt that the inflammation was located in the lung tissue. Figure 5b also shows a presence of a larger number of erythrocytes in rats exposed. In the pulmonary macrophages of the exposed rats (Fig. 6a) one can notice the pyrophyllite particles aggregates in the cytoplasm. At the same time, identical aggregates of the pyrophyllite particles were found in the cytoplasm of the pneumocytes (Fig. 6b) for the exposed rats.

These results are consistent with the work of Mistry et al. [28]. They have studied influence of inhalation of fluorescent carboxylated polystyrene (PS) nanoparticles of the size range 20, 100, and 200 nm in pigs. The observed changes were that the nanoparticles had penetrated the epithelium and migrated further to the blood and finally, had been detected in the brain tissue. The discussion in that study was first focused on the size-dependent effects, however, most probably the pH has also played an important role. Namely, they have used the buffer solutions in transferring the nanoparticles to the lung tissue, simulating the nasal liquids. The most importantly, a buffer of pH = 6 have shown a significant increase in transport from the donor chamber in Franz diffusion chamber experiments for the nanoparticles of 20 nm. Then, different results were also shown for other particles sized 80, 100 and 200 nm of different composition and at different pH. The first however, corresponds to the conditions of the study presented here in the case of pyrophyllite. It is important to reference the physico-chemical characterization part of the study given in Supplementary Table S2 These results show an extraordinary buffering potential of the pyrophyllite. It can be said with high certainty that the inhaled pyrophyllite in the present study has caused the similar buffering phenomenon to the values in proximity of 6–7 value of pH inside the lung secret. Therefore, it is possible that the pyrophyllite particles of smaller size have penetrated the epithelium in a similar manner like the polystyrene nanoparticles of around 20 nm diameter. In the technical report on pyrophyllite, the percent of the particles below 1 µm contributes less than 4% [9]. Therefore, it is possible that part of this size fraction has penetrated the epithelium in the present in vivo study on rats. The synergy of the present research and the research of Mistry, can now corroborate the study of Kishimoto et al. [29]. They performed an autopsy study of the lung tissue of two workers who worked for 15 and 11 years in the agalmatolites (pyrophyllite-containing ore) mine. Mineralogical examination in their cases clearly showed the presence of quartz, pyrophyllite, mica, and kaolinite which explained the histopathological examination of massive fibrosis, which corresponded to large shadows, but with only a small number of typical silicotic nodules in the lung. They concluded that it was a mixed dust pneumoconiosis. Connecting this study with the results shown here, it can be claimed with high certainty that the interaction between the nanoparticles of pyrophyllite and the pneumocytes starts even after a short exposure. This should be significant data even considering that the lifespan of a rat is much shorter than human and their body mass ratio much smaller comparing to human.

#### Liver tissue

**Fig. 7:**
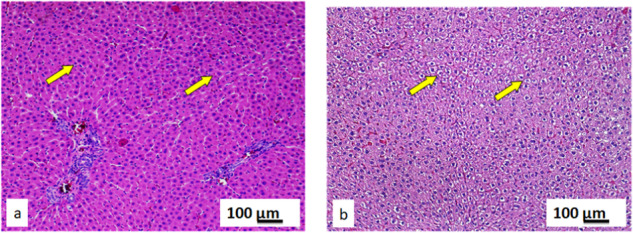
Micrographs of the histological cross-section of the liver, Mallory–Azan, magnification 50×, yellow arrows show distribution of blood sinusoids: control (**a**) and tissue exposed to pyrophyllite (**b**)

**Fig. 8:**
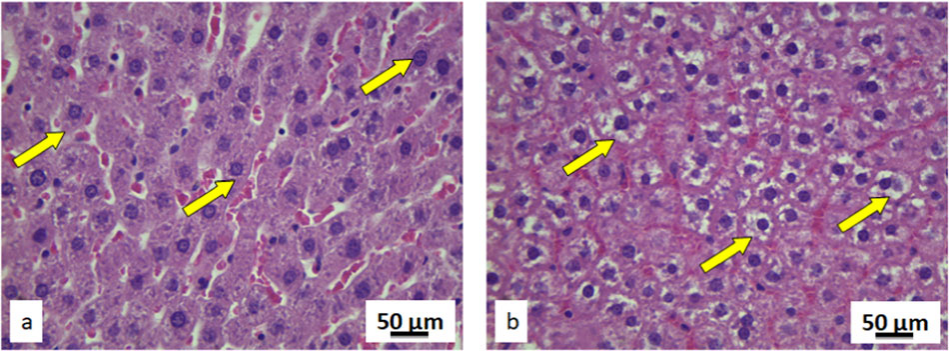
Micrographs of the histological cross-section of the liver, Mallory–Azan, magnification 100×, yellow arrows show hepatocytes with two nuclei: control (**a**) and tissue exposed to pyrophyllite (**b**)

Morphology of the level for the rats exposed to pyrophyllite, was not significantly different from the control. Hepatocytes and the blood vessel cells were of normal structure and positions in the exposed rats. Besides, their livers had no pathological changes like fibrosis, cysts, loss of hepatocytes, necrosis, and presence of lymphocytes or inflammatory processes. For this reason, the histological and cytological analysis of livers for both exposed and control groups were based on the comparison of 15 stereological parameters (Supplementary Table S6). This part of the results has shown that in the liver tissues of rats exposed to pyrophyllite, there is an increase in the volume and numerical density of hepatocytes and epithelial cells of blood sinusoids, as well as their mitotic index in relation to the control group. However, neither of these increases was significant. Liver cross-sections of the control group show normal distribution of blood sinusoid cells and red blood cells (Fig. 7a, yellow arrows). On the contrary, liver sections of rats exposed to pyrophyllite showed an increased distribution of the same histological elements (Fig. 7b, yellow arrows). The mitotic index of hepatocytes in control animals is within physiological limits (Fig. 8a), while the same parameter is elevated in rats exposed to pyrophyllite (Fig. 8b, row of arrows), which is a consequence of increased liver function.

Present results are valuable since they show the state of the liver as an organ that is always considered in all kinds of toxicological analysis. It performs the cleansing and the detoxification of the harmful substances which had eventually found their way to enter the organism. Liver tissue in the present study has shown no significant changes in the exposed group in comparison to the control group. Light microscopy did not reveal any presence of the macrophages or Kupffer cells with pyrophyllite particles in the liver tissue of exposed rats. This is completely opposite from the spleen tissue analysis discussed above, where the macrophages have been clearly identified. One can assume that probably the liver tissue had adapted to the presence of pyrophyllite and induced the self-regeneration. This conclusion is supported by the increased mitotic index in the present study. Similar data were obtained by Evans et al. in their study of NovaSil commercial clay in rats [1]. Also, other studies that included Clay 1, Clay 2 and Montmorillonite, concluded that the in vitro cytotoxicity of these clays on hepatocytes lines HepG2 was very low [24, 30]. The goal of their study was to access the safety of application of these clays in the food packaging and conclusion was that this application was an excellent and safe option. Here again, the study of montmorillonite as the drug carrier has reached the same conclusion [31]. They too, have shown that the clay does not accumulate in hepatocytes but there is a capillary dilatation and hepatocytes volume increase like in the present study, without accumulation of the clay particles in the hepatocytes. This is due to the increased liver activity.

#### Kidney tissue

**Fig. 9:**
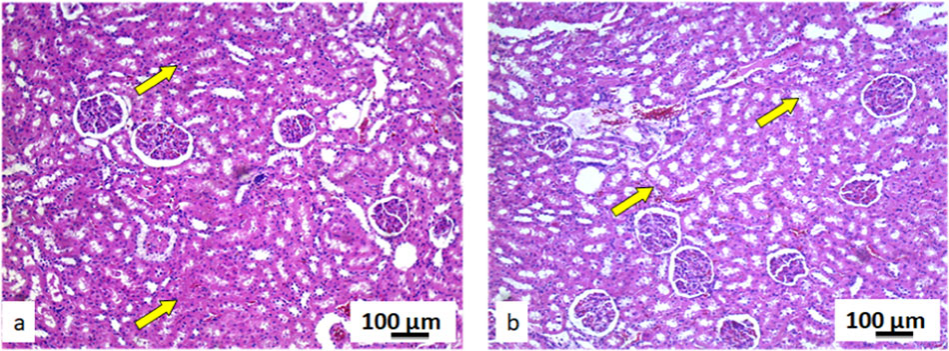
Micrographs of the histological cross-section of the kidney, H&E, magnification 50×, yellow arrows show decrease in the height of epithelia collecting ducts: control (**a**) and tissue exposed to the pyrophyllite (**b**)

**Fig. 10:**
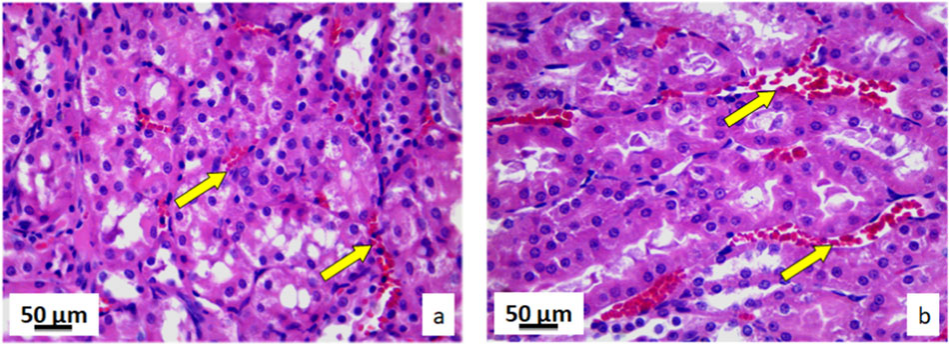
Micrographs of the histological cross-section of the kidney, H&E, magnification 100×: control, yellow arrows show position of capillaries (**a**) and tissue exposed to the pyrophyllite, yellow arrows show increase in the capillary volume (**b**)

**Fig. 11:**
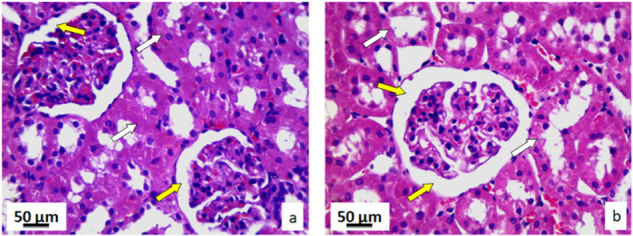
Micrographs of the histological cross-section of the kidney, H&E, magnification 100×, yellow arrows show Bowman’s space, white arrows show collecting ductus´ epithelial cells: control (**a**) and tissue exposed to the pyrophyllite (**b**)

In comparison to the control group, the kidney tissue of the exposed rats, had no morphological changes. Collecting ducts, kidney glomeruli and blood vessels had normal structure and position in the exposed group of rats. In this group, kidney tissue did not have and pathological changes such as cell loss in collecting ducts, loss of glomeruli, fibrosis, cysts, necrosis or presence of leukocyte aggregates or nodules. Furthermore, there were no infiltrates or inflammatory cells, inflammations, or edema in glomeruli. Histological and cytological analysis of the kidney tissue for both groups of rats was based on the comparison of 17 stereological parameters (Supplementary Table S7). Volume and numerical densities of the epithelial cells in the collecting ducts, as well as their surfaces were decreased with statistical significance (*p* < 0.05) in the exposed rats’ tissue comparing to the controls. On the other hand, volume and numerical density of the capillary epithelia cells, and their nucleocytoplasmic ratio were significantly increased (*p* < 0.05) in the exposed rat tissue. In the kidney tissues of rats exposed to pyrophyllite there was a decrease in the volume and numerical density of the epithelial cells of the collecting ducts, as well as an increase in the space between the Bowman’s capsule and the glomeruli in comparison to the control group. In the kidneys of rats exposed to pyrophyllite, there is a decrease in the height of the epithelial cells of the collecting ducts of the renal cortex (Fig. 9a, b) in relation to the control ones. The kidney cross-sections in the control group showed a normal distribution of blood sinusoid cells and red blood cells (Fig. 10a, yellow arrows), while the kidney cross-sections in rats exposed to pyrophyllite show (Fig. 10b, yellow arrows) an increased distribution of the same histological elements. In control animals, the size of the Bowman’s capsules and glomeruli were within the physiological limits (Fig. 11a, yellow), while in animals exposed to pyrophyllite, the size of the glomeruli was reduced and thus the space between it and the Bowman’s capsule (Fig. 11b, yellow arrow) was increased. Additionally, the height of epithelial cells in the collecting ducts of the marrow in the kidneys of rats exposed to pyrophyllite was reduced in comparison to control animals (Fig. 11b, white arrows).

Kidneys of exposed rats did not have histopathological changes and their activity and the blood flow collecting ducts were even increased. It was difficult to find any literature data on the influence of ingestion or exposure to the clay on kidneys. Regarding pyrophyllite, during preparation of the present manuscript, no literature at all was found in the electronically available databases. Present research revealed for the first time that, at least under the exposure conditions used here, there was no damaging effect to the kidneys nor the accumulation of the particles within the cells. The absence of such negative effects, can at least be useful in the recommendations of the this clay for food industry application, like for other clays [24]. Furthermore, authors working on other types of clays have found increased capacity of blood filtration through the collecting ducts, increase in blood flow and identical changes in the urine composition. They have also found an increase in urea, creatinine, and total proteins like in the present study on pyrophyllite (already discussed in section “Biochemical analysis” and given in Supplementary Table S4.). They stated that the reason for the increase in these three blood parameters was the higher activity of macrophages that are collecting the particles in their cytoplasm and get degraded inside the liver and the spleen [1, 32]. These observations completely apply to the present study as well they are matching the above findings revealing the presence of macrophages in the pulmonary and spleen tissue. Therefore, the same explanations on the kidney activity changes seem to be applicable.

#### Gastric tissue

The gastric tissue of rats exposed to pyrophyllite showed no morphological changes compared to the gastric tissue of rats from the control group. Epithelial cells and blood vessels of the gastric mucosa were of normal structure and position in rats exposed to pyrophyllite. No pathological changes in the tissue, such as inflammatory processes of the gastric mucosa, necrosis of enterocytes, cysts or the presence of leukocyte aggregates could be found in this group of animals. Histological and cytological analysis of gastric tissue of both groups of rats was based on the comparison of 15 stereological parameters (Supplementary Table S8). Volume density, number, numerical density, and mitotic index of endothelial cells (*p* < 0.05) increased significantly in the gastric tissue of rats exposed to pyrophyllite. These data, along with the clearly visible increased high of mucosa and number of enterocytes layers might be indication that pyrophyllite is acting as regeneration-inducing agent.

**Fig. 12:**
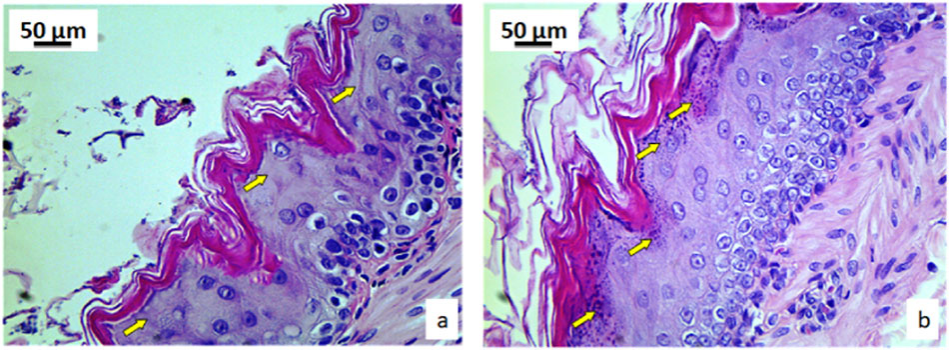
Micrographs of the histological cross-section of the gastric tissue, H&E, magnification 100×: control, yellow arrows are indicating enterocytes without pyrophyllite particles (**a**) and tissue exposed to the pyrophyllite with yellow arrows showing enterocytes with pyrophyllite particle aggregates (**b**)

**Fig. 13:**
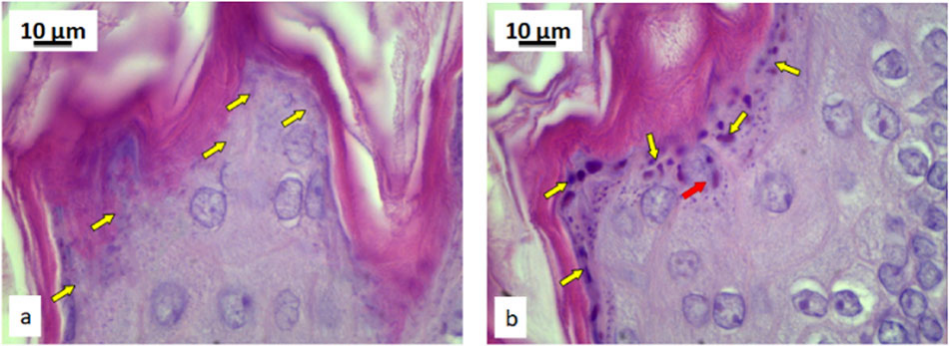
Micrographs of the histological cross-section of the gastric tissue, H&E, magnifications 250×: control, yellow arrows indicate mucosal surface without pyrophyllite aggregates (**a**) and tissue exposed to the pyrophyllite, yellow arrows indicate mucosal surface with pyrophyllite aggregates, while red arrow shows two large vesicles with pyrophyllite in the surface enterocytes (**b**)

Pyrophyllite can be easily observed on the surface of the mucous membrane of the exposed group, (Fig. 12b, yellow arrows) when compared with the control group (Fig. 12a). The last layer of mucosal enterocytes in the control rats does not contain pyrophyllite particles (Fig. 13a), while the same layer of enterocytes in the mucosa of rats exposed to pyrophyllite contains accumulations of pyrophyllite in their cytoplasm (Fig. 13b, red arrows).

It was not possible to find the comparative literature data on the exposure or enterocytes to pyrophyllite particles. However, it was shown in the case of polymer nanoparticles (50–200 nm) that the size and the surface charge had strong influence on their internalization into the cell membrane of the intestinal epithelial cells, while the shape of the particles did not influence this process [33]. Therefore, the ultrastructural TEM analysis was performed in the later phase in order to determine the size of the internalized particles.

### Ultrastructural analysis

**Fig. 14:**
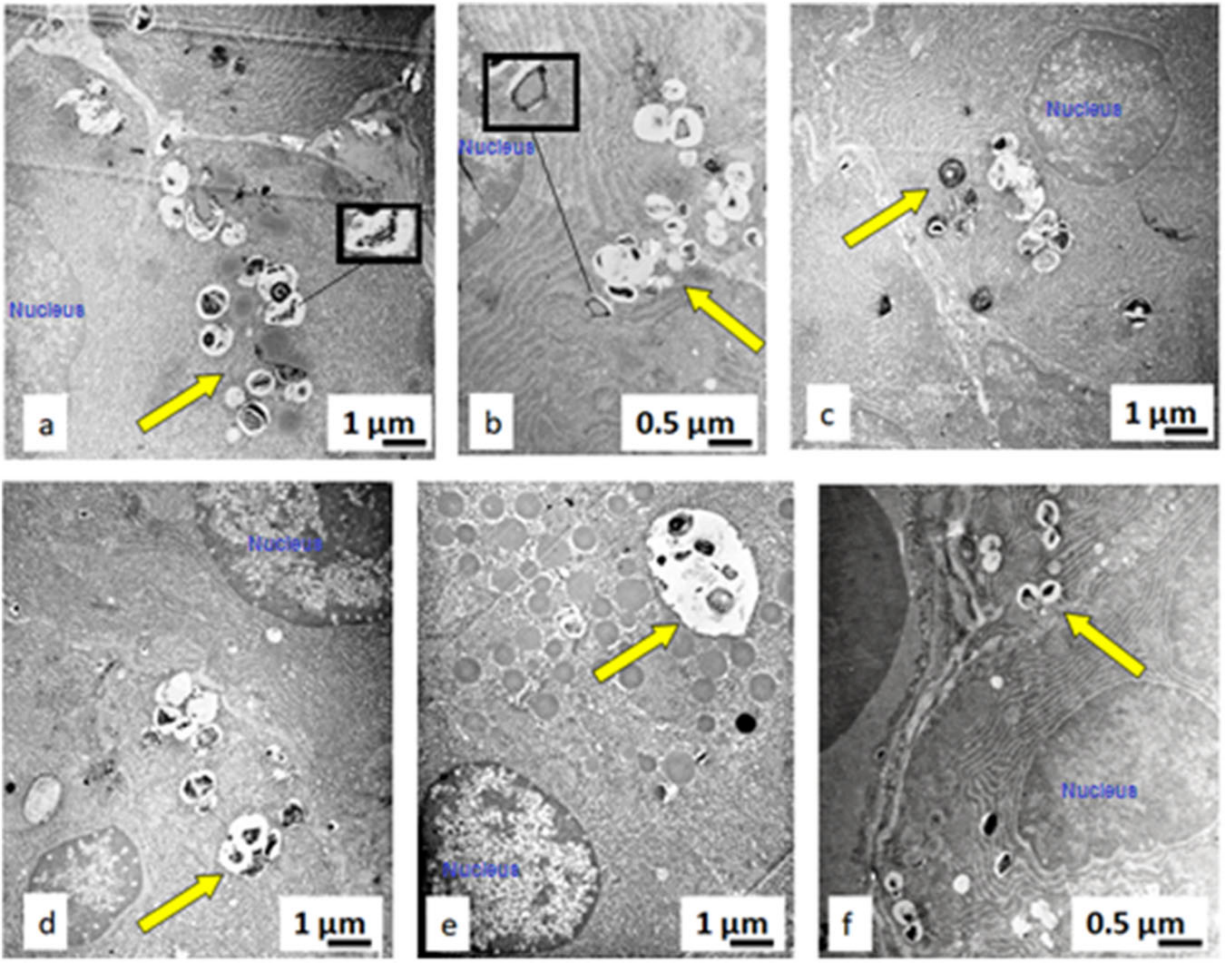
Transmission electron micrographs of the gastric tissue enterocytes at different locations. Yellow arrows depict endocytotic vesicles containing pyrophyllite particles

The ultrastructural analysis was used to study the interaction between the pyrophyllite particles and the enterocytes. Electron micrographs show enterocytes with pinocytotic vesicles that contain pyrophyllite particles, and this is evidence that pyrophyllite particles of a certain size range (cca. 50–500 nm) can enter gastric enterocytes (Fig. 14).

It can be said that these pyrophyllite nanoparticles were internalized into enterocytes during the feeding of animals with the pyrophyllite-containing food as described in the experimental setup. Thus, internalization of the particles was the consequence of the inability of the enterocytes to distinguish what the particles really were, since the positive charge of the pyrophyllite most probably have attracted the negative proteins from extracellular matrix, thus “tricking” the cell to get inside. It can be said with certainty, since the Fig. 13b shows that the easiest path for entrance was the apical surface of the enterocytes. This is in agreement with the results obtained in the studies aiming at applying this mechanism for drug delivery by both polymer nanoparticles and montmorillonite particles [31, 34]. The detailed investigation into the nanoparticles surface charge densities on their internalization by HeLa cells, has shown that the binding of the proteins to the gold nanoparticles is non-covalent, this kind of coating does not influence the surface charge of nanoparticle, it is the electrostatic attractions with the cells [35]. Literature cites that pyrophyllite has a point of zero charge of 1.97 which means that its surface is charged positively below 1.97 and negatively above [36]. Another source says that PZC values up to 4.2, suggesting PZC are also dependent of the ionic strength [37]. This paper also makes distinctions between PZC values between PZC of the edge and of planes, however, surface of pyrophyllite is for sure positive at least in the gastric tissue. The very acidic pH of gastric tissue would be favorable to positively charged pyrophyllite particles as also can be said from the fact that contact angle measurements confirmed a moderately hydrophobic pyrophyllite surface. It is also highly probable that the positive calcium ions (5.78 wt.% in the pyrophyllite structure) play an important role in this mechanism of internalization, especially after considering that this is one of the biomicronutrients. The most intriguing in the present results is the contrast between the fact that internalized pyrophyllite have caused an increase in mitotic index and volume density of enterocytes in the gastric tissue, stimulating the cell division and the fact that the particles remain trapped inside the endocytotic vesicles. Gastroprotective effects of some similar natural clays were confirmed by Ben Ali et al. These authors discussed the mechanism as the mechanical protective effect of the clay film on the gastric mucosa in relation to formation of ulcers induced by 95% ethanol [38]. However, Francia et al., in the extensive study of the mechanisms of the endocytosis of different nanomaterials, strongly insists that the main mechanism is the “corona effect” in which proteins bind to the nanoparticle surface and simulate the growth factor [39]. Strong evidence of the internalization of the pyrophyllite nanoparticles in present research, deserve further investigation and could point out to a new study area. It would be important to learn which exact protein, present in the gastric environment strongly adsorbs to natural clay nanoparticles and enables their endocytosis.

On the one hand there are many reports on the “corona-effect” which is substantially the formation of the layer of proteins around the nanoparticles which is relatively helpful in the process of the cell uptake of the particles. However, quite often, this corona-effect covers up and renders ineffective the functionalized moieties aimed at, for example, delivering some drug to the desired area in the tissue. This review also summarizes the other effects which are the uptake of the particles by one of the most common endocytotic mechanism which is the clathrin-mediated endocytosis. Besides this receptor protein, also some other proteins like dynein or dynamin, actin, kinesin, and other proteins can take part in endocytosis. To find out which exact receptor protein was involved in the present case of the endocytosis of the pyrophyllite, requires a separate study, however, it is very important to notice that the size-selectivity was present. Namely, less than 4% in the micronized material used in present study were nanosized pyrophyllite is contained in. Yet only the nanosized particles were internalized by the endocytotic mechanism. Clear evidence is visible from the transmission electron microscope images given in Fig. 14. Further the shells around the pyrophyllite nanoparticles are clearly visible in the inset images in the Fig. 14a, b, confirming the corona-structures, e.g. the shells of the proteins bound to the surface of pyrophyllite, which have most probably triggered the endocytosis.

## Conclusions

Pyrophyllite clay, showing all typical XRD peaks and presence of minerals, has shown excellent buffering properties towards pH around 8. Comet assay has shown complete absence of the genotoxicity of the pyrophyllite in human THP cells. Inhalation of pyrophyllite had distinctly negative effect on lung tissue characterized by a significant presence of macrophages, suggesting a strong inflammation.

Tolerability of pyrophyllite after oral administration was very good. Ultrastructural analysis of gastric mucosis has shown that the internalization was size-dependent, since only the 50–500 nm diameter particles were internalized by the corona effect of endocytosis. In future studies, it is necessary to learn if pyrophyllite particles maybe plays a role of epithelial growth factor in the gastric mucosal cells.

Overall value of the present research is in the detailed study of the biomedical potential of this rarely studied aluminosilicate, which is available as a natural resource at all world continents. Influence of the pyrophyllite on the gastric mucosa was studied for the first time and results go strongly in favor of the potential for biomedical application of pyrophyllite in the treatment of gastritis and similar diseases. Care must be first taken to the facts that certain leaching of lead, cadmium and chromium have been observed in physicochemical study, giving priority to the research on finding method for these metal traces before continuing the biomedical research and reaching the final comprehensive assessment of such processed pyrophyllite’s health implications [40]. These studies are on the way and are aligned with the already well-established methods in preparations of aluminosilicates for medical purposes and will be reported as the continuation of the present study.

## Methods

### Materials used

The pyrophyllite samples were taken at the deposit in Parsovići village, Konjic, Bosnia and Herzegovina (GPS coordinates: 43.773705, 17.811660). The ore was ground and separated to the average particle sizes ≤100 µm. Classification was performed in the professional ore preparation laboratory. Pyrophyllite micronisation was performed by the the standard laboratory ceramic ball mill, in the completely dried state. Samples were sieved to −100 μm and subsequently the particles size distribution was analyzed by laser diffraction on Malvern Mastersizer v3.50, in the water as a medium.

### Physicochemical characterizations of the pyrophyllite

The composition of pyrophyllite samples was determined by X-ray fluorescence (XRF) spectroscopy using a XEPOS spectrometer (Spectro Ametek). Samples were analyzed in their powder form and quantitative data were determined using the MicroPowder method combining the well-established fundamental parameters approach to XRF spectrometer calibration with automatic correction for matrix effects by using the Compton backscatter information from the sample to calculate the matrix interferences. X-ray diffractometry was performed in the region of 5–80° 2θ. Apparatus used was a D8 ADVANCE BRUKER X-ray diffractometer equipped with a CuKα-radiation source (wavelength 1.5406 Å, step 0.019 per min).

The metal leaching test was performed on the bases of the standard procedure [41]. The only modification was that the ratio of the liquid solid was increased from 10 to 100 for better suspension of the pyrophyllite and the pH values studied were more detailed than those ones demanded by this standard. Basically, for 24 h 1 g of pyrophyllite was shaken at 100 rpm in the 100 mL solutions of different pH, in the shaker bath at 25 °C. The pH of every solution was set to the values between 2 and 11, with nitric acid and sodium hydroxide used for pH regulations in acidic and basic regions, respectively. After addition of these solutions to the flasks containing pyrophyllite, and initial 1 min of shaking, the pH values were confirmed by means of the Hanna pH-meter model HI2002-02, and subsequently after 24 h before the filtration process. The powder fraction was then separated from the suspension by means of membrane filters of mash 0.45 µm.

After filtration through a standard filter paper black ribbon, the metal contents in the leachate were determined by digestion according to the literature means of the atomic absorption spectrometry model Analyst 400, Perkin Elmer, λ = 357.87 nm [42]. The focus of the study was on the potentially harmful toxic metals: Cu, Pb, Cd, Zn, Cr, Al, As and Hg.

### Genotoxicity studies in vitro

#### Cell exposure and viability evaluation

TPH-1 monocytic cells were purchased from Sigma-Aldrich, NZ (Cat. #88081200) and cultured in RPMI 1640 medium supplemented with 10% (v/v) FBS and 1% (v/v) penicillin/streptomycin (Invitrogen) at 37 °C in 5% CO_2_. Study was performed using monocytes according to the literature recommendations [43–45].Test THP-1 cells were seeded in 12-well plates in concentration 15 × 10^4^ cells per well. 24 h after seeding cells were exposed to pyrophyllite extract. Extract was prepared by immersion of cells in 10 mL of 20 mg/mL pyrophyllite solution in distilled water, with previously adjusted pH at 7.35, during 48 h. Pyrophyllite clay was previously sterilized at 105 °C for 2 h. The control THP-1 cells were not treated with pyrophyllite extract. Positive control was THP-1 cells exposed to methyl methanesulfonate solution (40 μM). The cells were exposed to emulsions of pyrophyllite for 1 h, after which they were centrifuged (200 g, 5 min) and resuspended in Dulbecco’s phosphate buffered saline (DPBS, Merck, Darmstadt, Germany). Trypan blue exclusion assay was used for THP-1 cell viability test [46, 47] after treatment with pyrophyllite and prior to alkaline Comet assay. Viability of THP-1 cells evaluation was performed by microscopic observation with Leica 8000D microscope with a MEGA VIEW camera, using a total magnification of ×400 and a digital transfer as well as photo analysis software system Application Suite 3.0.0. with minimum 200 cells/sample. Test was repeated 5 times for reproducibility.

#### Alkaline comet assay

Single cell gel electrophoresis assay namely Comet assay was used to detect possible single or double-stand DNA breaks, alkali labile site, and incomplete excision repair sites [47, 48]. Microscope slides for alkaline Comet assay were pre-coated with 0.5% normal-melting point (NMP) agarose, and test cells were mixed with 1% low-melting point (LMP) agarose and then applied onto slides. After agarose solidification, the additional layer of 1% LMP agarose was applied, and followed by the slide’s immersion in alkaline lysis solution for 60 min (1 M NaCl, 0.1 M EDTA, 10 mM Tris-HCl, 0.1% N-lauroylsarcosine, 1% Triton X-100, 30 mM NaOH). The slides were washed in distilled water and immersed for 20 min in cold electrophoresis buffer (0.3 M NaOH, 1 mM EDTA, pH > 13). After this, electrophoresis was applied (30 min at 0.7 V/cm) in the same buffer, and at electrophoresis, the slides were neutralized with 0.4 M Tris-HCl (pH 7.5) and stained with ethidium bromide (10 μg/ml). Analysis of slides was performed by using fluorescent microscope Leica DMi8 Fluorescence Imaging with a MEGA VIEW camera (total using magnification of ×400), and a digital transfer as well as photo analysis software system Application Suite 3.0.0. Comet assay was used to analyze 50 randomly selected nuclei per slide. Percentage of DNA presence in the comet’s tail (tail DNA %) was used as the DNA damage parameter.

### In vivo study

#### Animals

The study was performed in adult male Wistar rats weighing 350–380 g purchased from the Animal Department of the Faculty of Natural Sciences and Mathematics, University of Banja Luka, the Republic of Srpska, Bosnia and Herzegovina. The animals were given water and food *ad libitum*, kept at a temperature of 20–22 °C, with a 12 h light and darkness cycle. The final weight of the rats before euthanasia was ranging between 410–450 g. All animal procedures were following the Directive 2010/63/EU on the protection of animals used for experimental and other scientific purposes. The study was approved by the Ethics Committee on Animal Experiments at the Faculty of Natural Sciences and Mathematics, University of Banja Luka, Decision No 01-9-192.2/19, the Republic of Srpska, Bosnia and Herzegovina. Control of the health condition during the experiment was performed by the licensed surgical veterinarian.

#### Experimental protocol

Rats were divided randomly into two groups with 10 animals in each group. There were two rats per one cage for laboratory animals. The first, experimental group was fed with commercial food for laboratory animals which was additionally supplemented with the 4 wt. % of pyrophyllite clay. These rats were fed with pyrophyllite-enriched food during the following 6 weeks and each pair of rats consumed on average 312 g of this food weekly. Since pyrophyllite was falling off from the food onto their floor cloth it could not be excluded that some of microparticles were inhaled [49, 50]. Therefore, the histology of lungs was performed. The second group was a control group, fed with standard food for rats and they were not exposed to pyrophyllite in any form.

#### Blood analysis

At the end of the experiment the rats were put in general anesthesia (Ketamin 90 mg/kg body weight, Ketamine Hydrochloride Injection USP Rotexmedica-Germany in combination with Xylazine 5 mg/kg body weight 2% Xylazine, Cp Pharma, Bergdorf, Germany) and sacrificed by decapitation. The blood samples were taken from the lateral tail vein of all rats for hematological (red blood cells, mean cell volume, mean cell hemoglobin, mean corpuscular hemoglobin concentration, red blood cells distribution width, hemoglobin, hematocrit, white blood cells, lymphocytes, monocytes, granulocytes, platelet count, mean platelet volume) and biochemical analyses (total protein, triglycerides, cholesterol, glucose, creatinine, total bilirubin, alkaline phosphatase, aspartate aminotransferase (AST), alanine aminotransferase (ALT), glutamate piruvate transferase (GPT), gamma glutamate transferase (GGT), pancreas lipase, urea, potassium).

#### Histological and stereological analysis

After 6 weeks of all experimental treatments, all 20 rats were dissected. The rats’ livers, kidneys, stomachs and lungs were removed, immersed in Bouin’s solution for 24 h, for higher adsorption, further cut into smaller pieces and then immersed into fresh Buoin’s solution. Subsequently, dissected organs were prepared for light microscopy using the standard tissue preparation procedure. Samples of tissues of all organs were embedded in paraffin then cut in a frontal plane on a Leica rotary Microtome RM 2165 (Leica Mycrosystems, Wetzlar, Germany), in 5-μm-thick serial sections. Slices of all tissue were stained with hematoxylin-eosin (H&E) and Mallory-Azan (all stains by Merck KGaA, Darmstadt, Germany). Histology and stereological analyses were performed on every 5^th^ stained section in order not to repeat the analyzed structures. The qualitative histology analysis of the tissue slides was performed while using the light microscope a Leica DM8000 microscope with a MEGA VIEW camera and software system for digital image transfer, and magnification of ×50, ×100, ×250 and ×400. For quantitative, stereological analysis, micrographs were acquired in the RGB layout and converted to binary format. Measurements were made using a stereo-universal test system according to adapted Cavalier’s principle, with using 16.0 point-counting system (MBF software system Application Suite 3.0.0., MBF Bioscience, Williston, VT, USA), with P2 spacing grid at the maximum ×400 magnification. MBF software system was used to measure the surface and volume of all cells and nuclei, by the thickness of their diameters. Measuring the surface of cells and their nuclei enabled the measurement of the cells, cytoplasm, and nuclei volumes. The ratio between the cells’ nuclei and cytoplasm volume was used to determine the nucleocytoplasmic ratio (NCR). The mitotic index was determined at the ratio between the number of cells in mitosis and the total number of cells in 10 visible optical fields per slide, with maximum ×200 magnification [51–53].

#### Ultrastructural analysis

Representative parts of all isolated stomachs of rats from the experimental and control groups were cut under stereomicroscope into 1 × 1 mm samples. Five samples were taken from each stomach and first fixed for 0.5–1 min in Harrewald’s solution. Then, samples were transferred to a 2.5% glutaraldehyde solution in 0.1 M phosphate buffer (pH 7.2), and after 3 min, this solution was replaced with a new glutaraldehyde solution. Next, in these samples, ethanol was replaced by acetone, and they were embedded into epoxy resin. The ultra-thin section obtained by ultramicrotome Leica EM UC6 (Leica, Germany), and studied using Transmission Electron Microscopy (TEM) Zeiss EM902 at the high voltage of 80.0 kV. The ultra-thin sections were looked through first at a light microscope with small magnification ×500, and then the selected sections were more carefully studied under higher magnifications in the range of ×6000–18,000.

### Statistical analysis

All the values were presented as mean ± standard deviation. Group comparisons were performed while using parametric (two-way ANOVA, one-way ANOVA followed by Dunnett’s test and t-test) or nonparametric tests (Kruskal–Wallis and Mann–Whitney U-test), depending on data distribution, with significance set at *p* < 0.001 for blood analysis and biochemistry, while value was *p* < 0.05 for stereological analysis. Statistical software SPSS 20.0 (IMB corp., Armonk, NY, USA) was used for data processing.

## Supplementary Information

Supplementary material
