# Supplementary material for: Biocompatibility of nano/micro-sized pyrophyllite particles by pulmo, liver, kidney and gastric mucosis cells
Source: J Mater Sci Mater Med. 2024 Jun 17;35(1):30. doi: 10.1007/s10856-024-06793-z (PMC11182872; doi:10.1007/s10856-024-06793-z)
Supplement: Supplementary file 1 — Supplementary material [file 10856_2024_6793_MOESM1_ESM.docx]

**Genotoxicity and Tolerability of Nano/Micro-sized Pyrophyllite Particles by Liver, Kidney and Gastric Mucosis Cells**

Smiljana Paraš^1^, Jovana Paspalj^1^, Karima Baghdad^2,3^, Ognjenka Janković^4^, Miloš P. Stojiljković^4^, Ranko Škrbić^4^, Radoslav Gajanin^4^, Pascale Massiani^2^, Franck Launay^2^ and Suzana Gotovac Atlagić^1^*

1. University of Banja Luka, Faculty of Natural Sciences and Mathematics, Mladena Stojanovića 2, 78000 Banja Luka, Bosnia and Herzegovina
2. Sorbonne Université, Laboratoire de Réactivité de Surface (LRS – UMR 7197 CNRS), 4 place Jussieu, 72252 Paris Cédex 05 - France
3. University of Oran 1-Ahmed Ben Bella, Faculty of Exact and Applied Sciences, Materials chemistry laboratory-LCM, Department of Chemistry, P.O. Box 1524, Oran - 31005, Algeria
4. University of Banja Luka, Faculty of Medicine, Sime Mrkalja 14, 78000 Banja Luka, Bosnia and Herzegovina

| 1. Metal leaching per mass of the pyrophyllite, measured by atomic absorption spectroscopy |
| --- |

| **Starting pH** | **Metal leaching per mass of pyrophyllite** | | | | | | | |
| --- | --- | --- | --- | --- | --- | --- | --- | --- |
|  | **Cu** | **Pb** | **Cd** | **Zn** | **Cr** | **Al** | **As** | **Hg** |
|  | **(mg/kg)** | **(mg/kg)** | **(mg/kg)** | **(mg/kg)** | **(mg/kg)** | **(mg/kg)** | **(mg/kg)** | **(mg/kg)** |
| 2 | 113.00 | 289.00 | 18.00 | 112.00 | 100.00 | 442.90 | 0.30 | <0.1 |
| 3 | 69.00 | 110.00 | <10 | 73.00 | 76.00 | 245.60 | <0.1 | <0.1 |
| 4 | 79.00 | 118.00 | <10 | 132.00 | 84.00 | 53.80 | <0.1 | <0.1 |
| 5 | 54.00 | 96.00 | <10 | 103.00 | 72.00 | 29.40 | <0.1 | <0.1 |
| 5.5 | 64.00 | 57.00 | <10 | 73.00 | 12.00 | 52.70 | <0.1 | <0.1 |
| 6 | 69.00 | 57.00 | <10 | 127.00 | 84.00 | 35.60 | <0.1 | <0.1 |
| 6.5 | 34.00 | 19.00 | <10 | <0.00001 | <10 | 48.40 | <0.1 | <0.1 |
| 7 | 69.00 | 62.00 | <10 | 112.00 | 43.00 | 55.70 | <0.1 | <0.1 |
| 7.5 | 54.00 | <10 | <10 | 92.00 | <10 | 17.00 | <0.1 | <0.1 |
| 8 | 64.00 | 62.00 | <10 | 44.00 | 142.00 | 25.20 | <0.1 | <0.1 |
| 8.5 | 64.00 | <10 | <10 | 102.00 | 64.00 | 26.30 | <0.1 | <0.1 |
| 9 | 64.00 | 33.00 | <10 | 87.00 | 21.00 | 16.30 | <0.1 | <0.1 |
| 10 | 59.00 | <10 | 13.00 | 73.00 | 103.00 | 17.40 | <0.1 | <0.1 |
| 11 | 59.00 | 90.00 | 10.00 | 112.00 | 115.00 | 22.50 | <0.1 | <0.1 |
| 12 | 162.00 | 213.00 | <10 | 204.00 | 77.00 | 44.90 | <0.1 | <0.1 |

| 1. The changes in pH values during 24 h of metal leaching tests on pyrophyllite |
| --- |

| **Starting pH value** | **pH after 24 h of acid leaching** |
| --- | --- |
| 2 | 6.86 |
| 3 | 7.94 |
| 4 | 7.92 |
| 5 | 7.97 |
| 5.5 | 8.00 |
| 6 | 8.42 |
| 6.5 | 7.83 |
| 7 | 7.91 |
| 7.5 | 8.20 |
| 8 | 8.09 |
| 8.5 | 8.08 |
| 9 | 8.63 |
| 10 | 8.68 |
| 11 | 9.37 |

1. Results of blood analysis; value are shows as mean ± SD

| Parameters | Control  (n=10) | | | Pyrophyllite (n=10) | Reference range |
| --- | --- | --- | --- | --- | --- |
| Red blood cells (10^6^/mm^3^) | | | 9.25±1.84 | 8.66±1.49 | 7.27-9.65 |
| Mean cell volume (μm^3^) | | | 54.15±2.33 | 56.25±1.14 | 48.9-57.9 |
| Mean cell Hb (pg) | | | 17.48±1.12 | 19.24±0.69 | 17.1-20.4 |
| Mean corpuscular Hb concentration (g/dL) | | | 31.34±1.22 | 34.18±0.89 | 29.9-37.5 |
| Red blood cells distribution width (%) | | 15.55±2.52 | | 15.65±1.69 | 11.1-18.2 |
| Hemoglobin (g/dL) | | | 17.18±1.91 | 16.77±3.14 | 13.7-17.6 |
| Hematocrit (%) | | | 50.82±10.33 | 49.11±9.35 | 39.6-52.5 |
| White blood cells (10^3^/mm^3^) | | | 5.65±1.25 | 10.17±2.13* | 1.96-8.25 |
| Lymphocytes (10^3^/mm^3^) | | | 4.39±0.52 | 5.81±0.74 | 1.41-7.11 |
| Lymphocytes of WBC (%) | | | 77.75±15.79 | 57.19±14.13 | 55.6-86.3 |
| Monocytes (10^3^/mm^3^) | | | 0.18±0.07 | 0.09±0.11 | 0.03-0.18 |
| Monocytes of WBC (%) | | | 3.22±1.36 | 0.99±0.56 | 0.8-3.8 |
| Neutrophil granulocytes (10^3^/mm^3^) | | | 1.05±0.98 | 4.24±1.41* | 0.22-1.57 |
| Neutrophil gran. of WBC(%) | | | 18.75±2.11 | 41.78±9.32* | 6.2-26.7 |
| Eosinophil granulocytes (10^3^/mm^3^) | | | 0.02±0.01 | 0.02±0.01 | 0.01-0.16 |
| Eosinophil gran. of WBC (%) | | | 0.39±0.01 | 0.21±0.07 | 0.2-3.5 |
| Basophil granulocytes (10^3^/mm^3^) | | | 0.003±0.001 | 0.011±0.007 | 0-0.05 |
| Basophil gran. of WBC (%) | | | 0.05±0.02 | 0.10±0.09 | 0-0.8 |
| Platelet count (10^3^/mm^3^) | | | 648.16±343.38 | 564.58±320.45 | 538-1177 |
| Mean platelet volume (μm^3^) | | | 11.42±2.83 | 10.56±2.49 | 6.2-9.4 |

Reference values taken from ^31,32^, (*p<0.001; t-test).

1. Analysis results of biochemical parameters; the values are shows as mean ± SD.

| Parameters | Control  (n=10) | Pyrophyllite (n=10) | Reference range |
| --- | --- | --- | --- |
| Total protein (mg/dL) | 6.35±1.35 | 8.28±1.87* | 5.2-7.1 |
| Triglycerides - mg/dL | 44.34±6.65 | 52.85±8.14 | 8.7-60.7 |
| Cholesterol mg/dL | 45.16±7.38 | 42.21±7.11 | 14.4-87.6 |
| Glucose- mg/dL | 117.56±14.44 | 92.75±18.355 | 62.4-201.8 |
| Creatinine- mg/dL | 0.51±0.14 | 0.77±0.17 | 0.2-0.5 |
| Total Bilirubin - mg/dL | 0.073±0.012 | 0.126±0.054 | 0.05-0.15 |
| Alkaline Phosphatase - U/L | 171.44±28.74 | 99.51±19.45 | 62-230 |
| Aspartate Aminotransferase - U/L | 64.12±16.93 | 61.25±8.89 | 74-143 |
| Alanine Aminotransferase - U/L | 13.36±2.26 | 17.25±2.29 | 18-45 |
| Glutamate Piruvate Transferase - U/L | 47.53±16.92 | 60.08±26.79 | 24-55 |
| Gama Glutamate Transferase - U/L | 3.29±1.56 | 3.92±1.29 | 2-12 |
| Pancreas Lipase - mmol/L | 1.17±0.26 | 0.53±0.13 | 1-17 |
| Urea - mmol/L | 5.75±1.64 | 7.08±1.1 3 | 12.3-24.6 |
| Potassium - mmol/L | 3.11±0.38 | 7.47±0.95* | 3.82-5.55 |

Reference values taken from ^31,32^, (*p< 0.001; t-test)

1. Stereological parameters of the lung for the control and experimental groups of rats, values are shown as mean ±SD.

| Parameters | Control  (n=10) | Pyrophyllite  (n=10) |
| --- | --- | --- |
| Volume density of lung epithelial cells (mm^0^) | 0.309±0.054 | 0.423±0.044* |
| Volume density of capillary sinusoids (mm^0^) | 0.203±0.032 | 0.233±0.036 |
| Volume density of connective tissue (mm^0^) | 0.115±0.019 | 0.111±0.016 |
| Volume density of macrophages (mm^0^) | 0.009±0.001 | 0.083±0.012* |
| Number of lung epithelial cells | 182783.0±20414.6 | 223119.1±20986.5 |
| Numerical density of lung epithelial cells (mm^-3^) | 23446.7±2955.4 | 26785.3±2999.2 |
| Surface area of lung epithelial cells (μm^2^) | 136.4±5.5 | 142.5±8.3 |
| Surface area of lung epithelial cells nuclei (μm^2^) | 49.6±2.2 | 51.2±1.8 |
| Nucleocytoplasmic ratio of lung epithelial cells | 0.348±0.033 | 0.389±0.039 |
| Mitotic index of lung epithelial cells | 1.62±0.277 | 1.89±0.219 |
| Number of connective tissue cells | 93527.6±10014.7 | 90334.9±10056.8 |
| Numerical density of connective tissue cells (mm^-3^) | 11096.5±2579.1 | 11275.3±2335.6 |
| Surface area of connective tissue cells (μm^2^) | 98.6±4.7 | 96.3±4.9 |
| Number of capillary endothelial cells | 316609.3±29802.5 | 360781.5±27397.5 |
| Numerical density of capillary endothelial cells (μm^2^) | 56467.9±885.3 | 63972.5±1214.9 |
| Surface area of capillary endothelial cells (μm^2^) | 84.3±7.4 | 89.1±8.1 |
| Number of macrophages | 255.7±9.2 | 4462.8±342.6* |
| Numerical density of macrophages (mm^-3^) | 41.6±8.7 | 335.4±89.3* |
| Surface area of macrophages (μm^2^) | 231.4±10.1 | 223.6±14.2 |
| Surface area of macrophages' nuclei (μm^2^) | 97.4±4.6 | 99.8±5.2 |
| Nucleocytoplasmic ratio of macrophages | 0.378±0.024 | 0.424±0.029 |

(*p<0.05; t-test)

1. Stereological parameters of the liver for the control and experimental groups of rats, values are shown as mean ± SD.

| Parameters | Control  (n=10) | Pyrophyllite  (n=10) |
| --- | --- | --- |
| Volume density of hepatocytes (mm^0^) | 0.649±0.048 | 0.702±0.055 |
| Volume density of capillary sinusoids (mm^0^) | 0.159±0.007 | 0.173±0.011 |
| Volume density of connective tissue (mm^0^) | 0.122±0.006 | 0.123±0.008 |
| Number of hepatocytes | 281587.6±20189.4 | 342547.8±40317.1 |
| Numerical density of hepatocytes (mm^-3^) | 49386.3±2783.1 | 58402.7±4013.4 |
| Surface area of hepatocytes (μm^2^) | 150.2±3.2 | 149.4±2.8 |
| Surface area of hepatic nuclei (μm^2^) | 46.5±1.7 | 50.5±2.2 |
| Nucleocytoplasmic ratio of hepatocytes | 0.322±0.021 | 0.397±0.033 |
| Mitotic index of hepatocytes | 1.66±0.22 | 1.92±0.35 |
| Number of connective tissue cells | 128873.7±15445.2 | 113066.6±10252.2 |
| Numerical density of connective tissue cells (mm^-3^) | 22341.5±3687.9 | 20117.8±4136.3 |
| Surface area of connective tissue cells (μm^2^) | 100.1±4.5 | 103.4±2.9 |
| Number of capillary endothelial cells | 248553.4±29095.7 | 299685.3±32645.9 |
| Numerical density of capillary endothelial cells (mm^-3^) | 42725.5±1368.4 | 50837.1±2274.5 |
| Surface area of capillary endothelial cells (μm^2^) | 81.2±5.9 | 79.6±4.8 |

(*p<0.05; t-test)

1. Kidney stereological parameters of the control and experimental groups of rats, values are shown as mean ± SD.

| Parameters | Control  (n=10) | Pyrophyllite  (n=10) |
| --- | --- | --- |
| Volume density of collecting ductus' epithelial cells (mm^0^) | 0.348±0.049 | 0.289±0.049* |
| Volume density of blood sinusoids (mm^0^) | 0.256±0.032 | 0.308±0.041* |
| Volume density of connective tissue (mm^0^) | 0.118±0.009 | 0.114±0.009 |
| Volume density of glomeruli (mm^0^) | 0.278±0.042 | 0.289±0.041 |
| Number of collecting ductus' epithelial cells | 157683.9±18324.4 | 148843.5±11287.2 |
| Numerical density of collecting ductus' epithelial cells (mm^-3^) | 21034.2±3123.7 | 13653.1±4561.9* |
| Surface area of collecting ductus' epithelial cells (μm^2^) | 206.6±8.9 | 145.4±10.5* |
| Surface area of collecting ductus' epithelial cells nuclei (μm^2^) | 64.3±2.6 | 66.5±2.8 |
| Nucleocytoplasmic ratio of collecting ductus' epithelial cells | 0.265±0.024 | 0.378±0.035* |
| Number of connective tissue cells | 168371.5±20064.4 | 174237.7±23876.9 |
| Numerical density of connective tissue cells (mm^-3^) | 24522.9±3905.1 | 27890.4±3663.2 |
| Surface area of connective tissue cells (μm^2^) | 103.5±6.1 | 101.1±11.4 |
| Number of capillary endothelial cells | 289345.2±20164.5 | 314572.8±25861.2 |
| Numerical density of capillary endothelial cells (mm^-3^) | 26093.4±3418.2 | 41325.5±3970.1* |
| Surface area of capillary endothelial cells (μm^2^) | 74.3±9.9 | 75.9±8.9 |
| Surface area of glomeruli (μm^2^) | 4253.5±311.5 | 3576.2±217.4 |
| Bowman's space | 43.9±3.5 | 50.1±2.9 |

(*p<0.05; t-test)

1. Stereological parameters of the gastric for both groups of rats, values are shown as mean ± SD.

| Parameters | Control  (n=10) | Pyrophyllite  (n=10) |
| --- | --- | --- |
| Volume density of enterocytes (mm^0^) | 0.364±0.041 | 0.491±0.075* |
| Volume density of capillary sinusoids (mm^0^) | 0.175±0.028 | 0.208±0.035 |
| Volume density of connective tissue (mm^0^) | 0.092±0.009 | 0.083±0.009 |
| Number of enterocytes | 98201.4±9014.3 | 141512.3±9950.6* |
| Numerical density of enterocytes (mm^-3^) | 7423.9±838.1 | 11885.3±1245.7* |
| Surface area of enterocytes (μm^2^) | 324.7±24.3 | 299.2±20.1 |
| Surface area of enterocytes' nuclei (μm^2^) | 63.3±2.5 | 56.6±2.1 |
| Nucleocytoplasmic ratio of enterocytes | 0.283±0.041 | 0.311±0.044 |
| Mitotic index of enterocytes | 1.59±0.255 | 2.99±0.343* |
| Number of connective tissue cells | 117233.3±9897.4 | 118884.2±8704.1 |
| Numerical density of connective tissue cells (mm^-3^) | 12098.3±1451.5 | 14403.6±2909.3 |
| Surface area of connective tissue cells (μm^2^) | 85.3±4.2 | 88.5±5.1 |
| Number of capillary endothelial cells | 205673.2±35872.5 | 224453.7±40924.1 |
| Numerical density of capillary endothelial cells (mm^-3^) | 19252.5±1774.4 | 20178.3±1800.5 |
| Surface area of capillary endothelial cells (μm^2^) | 86.4±3.3 | 88.5±4.7 |

(*p<0.05; t-test)
